# Parents’ burden of adolescents with autism spectrum disorder and coping mechanisms: which association?

**DOI:** 10.1192/j.eurpsy.2025.1148

**Published:** 2025-08-26

**Authors:** R. Jbir, F. Cherif, R. Masmoudi, N. Bouattour, J. Boudabous, M. Chaabene, I. Feki, F. Guermazi, J. Masmoudi

**Affiliations:** 1 psychiatry ‘A’ department; 2 Child psychiatry department, Hedi Chaker university hospital, Sfax, Tunisia

## Abstract

**Introduction:**

Adolescence in young people with autism spectrum disorder (ASD) is a special time of life, for them facing physical and mental changes, and for their parents who must cope with the new challenges associated with their child’s development. Research has shown that the severity, violence and behavioral disorders help to explain the major difficulties encountered by these families.

To cope with these difficulties, parents often use a variety of coping strategies. These coping strategies encompass all an individual’s cognitive and behavioral efforts, which are constantly evolving, to manage a situation perceived as stressful.

**Objectives:**

Our study aimed to assess the coping strategies adopted by parents of adolescents with ASD, their level of burden and to determine the relationship between these two variables

**Methods:**

We conducted a descriptive and analytical cross sectional study among parents of adolescents with ASD followed at the “Erraihan” therapeutic farm in Sfax during the period of May 2024.

The hetero-questionnaire used a pre-established form for collecting data relating to parents and adolescents and two psychometric scales:

- The BRIEF-COPE scale: to assess coping mechanisms in parents, of which there are four: problem-focused mechanisms, avoidance-focused mechanisms, social support-focused mechanisms and emotion-focused mechanisms.
- The zarit Burden Inventory (ZBI): used to assess the level of burden.

**Results:**

Forty-three parents were enrolled. Their mean age was 50.6 ±7.93 years (min=36; max=81). The average age of the adolescents was 17.79 years, with extremes of 13 and 20 years.

Emotional focus (mean score=2.54) was the coping mechanism most used by parents, followed by problem focus (mean score=2.50) and social support (mean score=2.45).

Avoidance was the least-used coping mechanism (mean score=1.63). Assessment of parental burden showed that the mean Zarit score was 24.6±20.14, with extremes of 0 and 48. Twenty-seven parents (62.80%) felt a burden of severe intensity.

In our study, only the humor-based emotion-focused coping strategy was more important in parents with absent to moderate levels of burden (1.98 versus 1.41; p=0.02). For the other coping strategies, there was no significant association with level of burden. Social support scores between parents with absent to moderate and severe levels of burden were close.

**Conclusions:**

Our results show that parents feel the heavy burden of caring for their sick adolescent. Indeed, ASD generates a great deal of stigmatization in the parent, who avoids discussing the illness with those around him, for fear of suffering further stigmatization. This vicious may explain the absence of a link between the level of burden and coping strategies centred on social support.

Thus, the question of the role of family support and associations for autistic families, proves crucial.

**Disclosure of Interest:**

None Declared

